# Therapeutic Remodeling of the Tumor Microenvironment Enhances Nanoparticle Delivery

**DOI:** 10.1002/advs.201802070

**Published:** 2019-01-22

**Authors:** Yuanxin Chen, Xiujie Liu, Hengfeng Yuan, Zhaogang Yang, Christina A. von Roemeling, Yaqing Qie, Hai Zhao, Yifan Wang, Wen Jiang, Betty Y. S. Kim

**Affiliations:** ^1^ Department of Neurosurgery Mayo Clinic 4500 San Pablo Rd Jacksonville FL 32224 USA; ^2^ Department of Orthopaedic Surgery Zhongshan Hospital Fudan University No. 180 Fenglin Rd Shanghai 200032 China; ^3^ Department of Chemical and Biomolecular Engineering Ohio State University 151 W. Woodruff Ave. Columbus OH 43210 USA; ^4^ Department of Neurosurgery West China Hospital No. 37th Guoxue Xiang Chengdu Sichuan 610041 China; ^5^ Department of Radiation Oncology University of Texas Southwestern Medical Center 2280 Inwood Rd Dallas TX 75390 USA

**Keywords:** DC101, extracellular matrix, nanomedicine delivery, normalization, TGFβ, tumor vasculature

## Abstract

A major challenge in the development of cancer nanomedicine is the inability for nanomaterials to efficiently penetrate and deliver therapeutic agents into solid tumors. Previous studies have shown that tumor vasculature and extracellular matrix regulate the transvascular and interstitial transport of nanoparticles, both critical for successfully delivering nanomedicine into solid tumors. Within the malignant tumor microenvironment, blood vessels are morphologically abnormal and functionally exhibit substantial permeability. Furthermore, the tumor extracellular matrix (ECM), unlike that of the normal tissue parenchyma, is densely packed with collagen. These pathophysiological properties greatly impede intratumoral delivery of nanomaterials. By using an antivascular endothelial growth factor receptor antibody, DC101, and an antitransforming growth factor β1 (TGF‐β1) antibody, normalization of the tumor vasculature and ECM is achieved, respectively, in a syngeneic murine glioma model. This normalization effect results in a more organized vascular network, improves tissue perfusion, and reduces collagen density, all of which contribute to enhanced nanoparticle delivery and distribution within tumors. These findings suggest that combined vascular and ECM normalization strategies can be used to remodel the tumor microenvironment and improve nanomedicine delivery into solid tumors, which has significant implications for developing more effective combinational therapeutic strategies using cancer nanomedicine.

One of the major limitations of cancer nanomedicine is the inefficient delivery of nanomaterials into solid tumors.^1^ Although nanoparticles preferentially accumulate within tumor tissue to a greater extent than in normal tissue owing to enhanced permeability and retention effect,^2^ the abnormal and dysfunctional tumor microenvironment often results in the heterogeneous distribution of nanoparticles, which predominately reside in the perivascular area and tumor periphery.^3^ Several pathophysiological features intrinsic to tumors contribute to impeding nanoparticle and macromolecular transport into and within solid tumors.^4^ First, unlike blood vessels within normal tissues, tumor vessels are morphologically abnormal and functionally impaired.^5^ Tumor vasculatures have a chaotic and irregular appearance and they also exhibit increased permeability due to lack of proper pericyte coverage and decreased perfusion capacity. This increased permeability is a direct result of excessive production of proangiogenic growth factors such as vascular endothelial growth factor receptor‐2 (VEGFR‐2), leading to the formation immature vessels that are unable to maintain adequate perfusion of tumor tissues.^6^ Second, within the tumor interstitial space, elevated expression of cytokines such as TGF‐β1 by both tumor and stromal cells promotes collagen synthesis, leading to the formation of a fibrous extracellular space.^7^ The dense ECM restricts diffusion and acts as a “trap” that further limit nanoparticle infiltration into the tumor parenchyma after extravasation.^8^ Significant research over the years has focused on designing better nanomedicine platforms to enable more efficient and uniform delivery into solid tumor.^9^ However, methods that prime the tumor microenvironment to render it more favorable toward nanomedicine delivery have been less investigated, and represent a novel approach to formulate more effective combination strategies for cancer nanomedicine.

In this study, we hypothesized that simultaneously normalizing the tumor vessels and ECM would facilitate the delivery and distribution of nanoparticles into solid tumors. By using a syngeneic malignant glioma tumor model, we set out to determine whether a combined anti‐VEGFR and anti‐TGF‐β1 treatment could significantly normalize tumor vasculatures and reduce collagen density within the ECM. Using multiphoton imaging, our goal is to determine the effect of simultaneous tumor vascular and ECM normalization on nanoparticle delivery and distribution with the tumor interstitium in vivo, thereby providing a strong preclinical rationale for combining a microenvironmental priming strategy with nanomedicine for cancer treatments.

We previously studied the effect of vascular normalization on improving nanoparticle delivery in murine syngeneic breast cancers, and found that the optimal intratumoral nanoparticle delivery relies on both transvascular and interstitital transport mechanisms, dictated by the tumor vasculature network and ECM, respectively.^10^ Normalizing the tumor vasculature did not improve the interstitial delivery of nanoparticles once they entered the tumor parenchyma, as it is subjected to diffusional hindrance by the ECM.^10^ To evaluate whether simultaneous normalization of the tumor vasculature and ECM can improve intratumoral delivery of nanoparticles into and within tumors, we established an orthotopic syngeneic glioblastoma model via stereotactic implantation of GFP or non‐GFP labeled murine GL261 cells. Using intravital two‐photon microscopy via a transparent cranial window, we found the brain tumor vasculatures are highly disorganized and chaotic, and exhibit significantly increased permeability as compared to normal brain vessels (**Figure** **1**a,b). Further, compared to normal brain parenchyma, the extracellular compartment of brain tumors was densely packed with collagen (Figure 1c,d), which when combined with increased cellular mass and elevated interstitital fluid pressure, compressed intratumoral vessels, and impede blood flow (Figure 1e; Figure S1, Supporting Information; Movies S1 and S2, Supporting Information).

**Figure 1:**
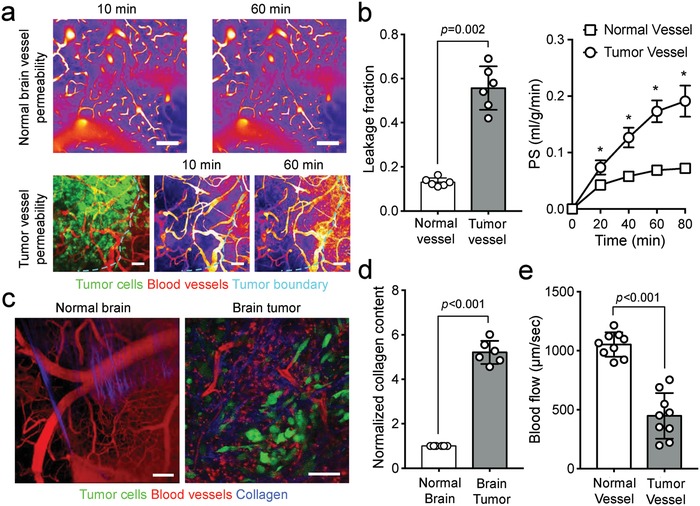
Brain tumors harbor abnormal vasculatures and extracellular matrix. a) Compared to normal brain blood vessels, tumor vessels are chaotic, heterogeneous, and irregular. The morphologically abnormal blood vessels are b) more leaky, due to increased vascular permeability. PS = permeability surface‐area product, *n* = 5. c) Furthermore, compared to normal brain tissues, brain tumors are densely packed with collagen. d) The significantly increased collagen content within the tumor, combined with elevated interstitial fluid pressure and cellular mass, compresses intratumoral vessels, leading to e) blood flow stasis. * denotes *p* < 0.05; error bars = mean ± standard deviation. Scale bars = 100 µm.

To characterize how anti‐VEGFR and anti‐TGFβ1 treatments can normalize brain tumor vasculatures and ECM, respectively, we treated C57BL/6 mice orthotopically implanted with GL261 glioma with isotype‐matched IgG, a rat anti‐VEGFR‐2 antibody (DC101; 10 mg kg^−1^ every 3 days), or a murine anti‐TGFβ1 antibody (100 µg every 3 days) (**Figure** **2**a). By using intravital microscopy, we observed DC101 progressively restored tumor vessels by reducing their tortuosity and density beginning 2 days after treatment initiation, and by day 8, vessel regression became more noticeable (Figure 2b,c). The structural changes to tumor vessels were accompanied by improved pericyte coverage, as measured by the proportion of NG2^+^CD31^+^ vessel regions, leading to a decrease in vascular leakiness (Figure 2d). Unlike blood vessels in other parts of the body, the permeability of central nervous system (CNS) vasculatures is also regulated by the specialized blood‐brain‐barrier. For CNS vessels, the permeability surface‐area product (PS) has been used as a marker for blood‐brain‐barrier permeability.^11^ We found that PS was highest in IgG‐treated group, indicting more plasma efflux from vessels, and dropped significantly in the DC101‐treated groups. These structural and functional alterations to tumor vessels in response to DC101 is consistent with the vascular normalization effect of VEGFR blockade reported in other tumor models.^12^

**Figure 2:**
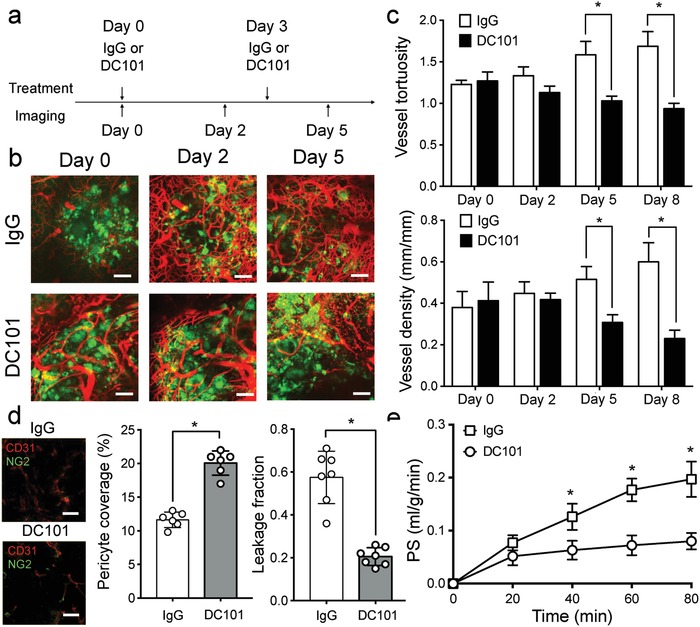
DC101 treatment normalizes tumor vasculature and improved vessel functions. a) Schematics demonstrate the treatment schedule for DC101 and anti‐TGFβ. b) Intravital microscopy revealed that DC101 normalizes blood vessels in orthotopically implanted GL261 glioma as compared to iso‐matched IgG control. Green = GFP labeled tumor cells. Red = DsRed‐dextran labeled tumor vasculatures. c) DC101 treatment resulted in morphological normalization of tumor vessels as measured by decreases in vessel tortuosity and density as early as 5 days after treatment initiation. d) The normalized tumor vessels also exhibit more mature phenotype as demonstrated by increased pericyte coverage (NG2^+^CD31^+^/CD31^+^), leading to a decrease of vascular leakage and e) permeability. * denotes *p* < 0.05; error bars = mean ± standard deviation; *n* = 7. PS: permeability surface‐area product. Scale bars = 100 µm.

We next evaluated the effect of anti‐TGFβ antibody treatment on collagen density within the tumor ECM. TGFβ1 is a well‐known inducer of collagen deposition and promoter of stromal cell differentiation.^13^ It also regulates the activities of matrix metalloproteinases and is thus critical for ECM remodeling in tumors.^14^ Treating GL261 tumors with an anti‐TGFβ antibody significant reduced the density and the overall collagen content within the tumor matrix (**Figure** **3**a). This decrease was significant as soon as day 2 after the first treatment dose (Figure 3b). Given that VEGF can directly modulate TGFβ signaling in endothelial cells,^15^ we next assessed whether DC101 treatment alone would also induce changes in ECM collagen. As expect, low dose DC101, although sufficient to promote tumor vessel normalization, did not result in noticeable decrease in the collagen content within the glioma ECM (Figure S2, Supporting Information).

**Figure 3:**
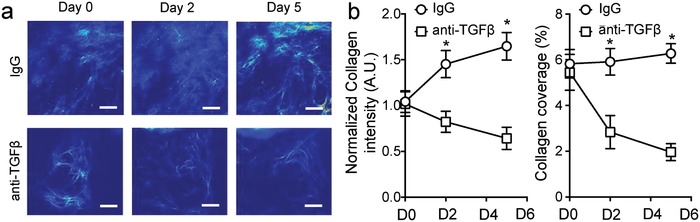
Anti‐TGFβ normalized the tumor ECM. a) Representative intravital 2‐photon microscopy images showing collagen matrix using second harmonic generation (SHG). b) Treatment with murine anti‐TGFβ antibodies significantly decreased the density of collagen in GL261 glioma. Significant decreases in ECM collagen content were observed as early as 2 days after treatment initiation. * denotes *p* < 0.05; error bars = mean ± standard deviation; *n* = 7. Scale bars = 100 µm.

Next, to test whether the normalization of tumor blood vessels and ECM can improve intratumoral nanomedicine delivery, we treated tumor‐bearing mice with combined DC101 and anti‐TGFβ1 antibody and measured the distribution of nanoparticles within the tumor interstitium. We used water‐soluble semiconductor nanocrystals (QD) with surfaces modified by 10KD molecular‐weight polyethylene glycol (PEG) as fluorescence tracers. These monodispersing nanoparticles possess a final hydrodynamic diameter of ≈40 nm, with a slightly negative surface charge (Figure S3, Supporting Information) and also exhibit a high degree of photo‐ and physical stability within biological environments (Figure S4, Supporting Information), thus enable us to perform quantitative studies on particle accumulation using fluorescence intensity measurements. We found that the combined DC101 and anti‐TGFβ1 antibody treatment significantly enhanced the intratumoral delivery of the fluorescence nanoparticles, resulting in deeper tissue penetration and a more homogeneous distribution of these particles within the tumor interstitium (**Figure** **4**a,d,e). We also noted that although DC101 treatment alone could increase the tissue penetration depth of 40 nm nanoparticles and improved their distribution profile (Figure 4b,d), the combination treatment allowed the nanoparticles to reach tumor areas twice as far as the closest perfusing vessel (Figure 4b). This improved tissue access by nanoparticles allowed them to accumulate more uniformly within the tumor interstitium (Figure 4a,e). Since both transvascular and interstitial transport of nanoparticles within tumors are highly dependent on their sizes, we next evaluated whether the improved tissue delivery effect also applied to larger sized nanoparticles. Given that a 100 nm size is more representative of currently approved nanomedicines (e.g., Abraxane) for cancer treatment, size in clinical trial, we next investigated whether combined DC101 and anti‐TGFβ1 antibody treatment enhance the distribution and penetration of 100 nm PEGylated polystyrene (PS) nanoparticles within the tumor. We found that, compared to IgG treatment, DC101 or anti‐TGF‐β monotherapies did not improve the penetration of 100 nm PS nanoparticles into the tumor tissue after intravenous injection. However, combining DC101 with anti‐TGF‐β antibody extended the tumor penetration and coverage of 100 nm nanoparticles by more than twofold (Figure 4d). The improvements, although significant, were nevertheless much less than that of 40 nm nanoparticles, suggesting that intermediate sized nanoparticles are likely to benefit most from tumor microenvironment normalization strategies aimed at improving nanomedicine intratumoral delivery. Interestingly, when we measured the total uptake of the nanoparticles within the tumor, the combined DC101 and anti‐TGFβ1 antibody treatment did not result in an increase in the unit accumulation of nanoparticles as compared to DC101 treatment alone (Figure S5, Supporting Information). These observations suggest that the normalization of the tumor vessels and ECM do not increase the total nanoparticle delivery into the tumor per se, but rather helps to more evenly distribute the extravasated nanoparticles within the intratumoral space.

**Figure 4:**
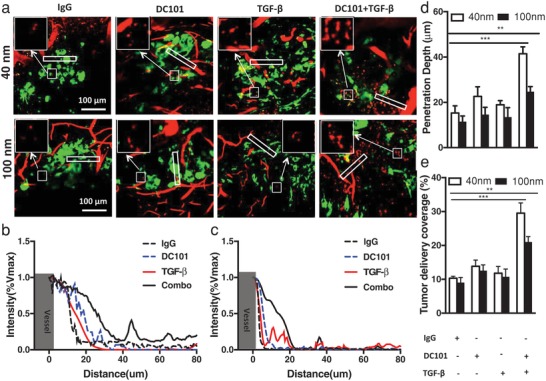
Combined anti‐VEGFR (DC101) and anti‐TGFβ treatment resulted in improved intratumoral delivery of nanoparticles. a) Sample intravital microscopy images showing combined treatments significantly improved nanoparticle penetration into the tumor parenchyma. Green = GFP labeled tumor cells. Red = Fluorescent nanoparticles. Measurement of fluorescence intensity showed improved penetration of b) 40 nm and c) 100 nm, respectively, as combined to IgG or DC101 alone. The combined DC101 and anti‐TGFβ treatment resulted in more uniform delivery and deeper penetration of nanoparticles within the tumor interstitium as compared to IgG, DC101 alone or anti‐TGFβ treatment alone. The combo treatment enhanced d) penetration and e) uniform distribution of 40 and 100 nm within tumor area. Scale bar = 100 µm; * denotes *p* < 0.05; error bars = mean ± standard deviation; *n* = 7.

Achieving efficient and uniform delivery of nanomedicine into solid tumors has been a major challenge facing cancer nanotechnology research. A recent metaanalysis further suggested that the inability to more effectively deliver nanomedicine from systemic circulation into the tumor parenchyma has severely impeded its potential for clinical translation.^1^ Various strategies have been used over the years to design nanoparticles that can efficiently escape nonspecific clearance by the body's mononuclear phagocytosis system or can alter their size and surface charges according to environmental stimuli to minimize transport hindrance.^9^ Similarly, multistage and multipurpose nanoparticle systems have also been developed to provide better tissue penetration without compromising drug payload.^16^ However, to date these nanoparticle‐centered strategies to improve intratumoral delivery have provided only modest benefits.

Abnormalities in the tumor microenvironment result in pathophysiological features that prohibit the efficient transport of nanomedicine and macromolecules into the interstitial space of the tumor.^17^ Tumor vessels are immature and have a highly chaotic and heterogeneous vascular network. The lack of pericyte coverage around the endothelial walls makes tumor vessels more leaky to macromolecules and proteins, leading to an increase in the oncotic pressure within the interstitial space. Within the tumor microenvironment, interstitial fluid pressure (IFP) is elevated at the tumor center and drops rapidly at the tumor margin. This abnormal pressure distribution is a direct result of the excessive leakiness of tumor vessels, which are unable to maintain a pressure gradient across the vascular walls, causing the IFP to reach levels equivalent with the microvascular pressure.^18^ Elevated IFP also causes flow stasis by compressing intratumoural vessels, leading to decreased tissue perfusion, further hypoxia, and increased production of proangiogenic factors. This vicious cycle continually fuels abnormalities within the tumor microenvironment. As a consequence, therapeutic agents and nanoparticles face significant resistance entering the tumor and are preferentially carried away toward the tumor periphery.^18^

Even if nanoparticles are able to extravasate from the intraluminal space of blood vessels into the tumor interstitium, their transport is further restricted by diffusional hindrance, exerted by a densely packed ECM.^10^ Previous studies have shown that modification of the ECM by degrading collagen or hyaluronan improved the diffusional transport of macromolecules such as IgG within solid tumors.^19^ However, delivery of matrix‐degrading enzymes can only be done locally via intratumoral injection and is therefore not practical in most clinical scenarios.

Here, we investigated whether combined normalization of tumor vessels and ECM could result in improved delivery of nanoparticles into solid tumors, leading to a more uniform distribution pattern. We demonstrate that by using low‐dose anti‐VEGFR and anti‐TGFβ1 antibodies, we could normalize blood vessels and decrease collagen content within murine syngeneic gliomas. These physiologic and structural changes in the tumor microenvironment increased the depth of penetration of nanoparticles and the uniformity of their distribution within the tumor. Notably, although TGFβ has long been established as a potent inducer of collagen synthesis and differentiation of stromal cells such as fibroblasts, recent studies have found that TGFβ inhibition in itself can lead to a decrease in pericytes coverage, resulting in enhance tumor vessel permeability in pancreatic adenocarcinoma.^20^ These findings further highlight the multifunctional and heterogeneous role of TGFβ in tumorigenesis in different tumor models. A number of studies have demonstrated that TGFβ signaling promotes glioma angiogenesis via the upregulation and activation of various angiogenic factors including VEGF.^21^, ^22^ In our study, adding anti‐TGFβ antibody did not result in an increased antiangiogenic effect when compared with DC101 treatment alone, thus confirming that the proangiogenic function of TGFβ is likely to be predominantly mediated via VEGF.

Finally, it is important to realize the normalization of both tumor vessels and ECM is a transient event that is strongly dictated by the dose and duration of the therapeutic agents given and by the characteristics of the tumor itself.^10^, ^23^, ^24^ Investigations have already been conducted to explore vascular normalization strategies beyond disrupting the VEGF/VEGFR axis. Inhibition of angiopoietin‐2 (ANG2), for example, has been found to promote more durable tumor vessel normalization when combined with anti‐VEGF treatment relative to VEGF blockade alone in glioblastoma.^25^, ^26^ Therefore, an optimized tumor microenvironment normalization strategy to improve the delivery of nanomedicine and macromolecules will likely require simultaneous targeting of multiple aberrant pathways involved in the pathophysiological transformation of the tumor, and must be tailored to specific tumor type of interest.^27^ Nevertheless, tumor microenvironment priming strategies represent unique opportunities that in combination with nanoparticle designs could achieve most efficient delivery of nanomedicine into solid tumors.

The present study suggests that simultaneous normalization of abnormal tumor vasculature and components of the ECM can enhance the delivery of nanomedicine into solid tumors. The finding that the combined anti‐VEGFR and anti‐TGFβ treatment resulted in enhanced and more uniform accumulation of nanoparticles within the tumor interstitium relative to either therapy alone further confirms that intratumoral transport of nanoparticles and macromolecules is a two‐step process, governed by distinctive cellular and pathophysiological properties of the tumor. The development of tumor microenvironment modulation strategies to improve the transvascular and interstitial transport of nanoparticles, coupled with a more complete understanding of how intrinsic properties of nanomaterials affect intratumoral delivery, can pave the way for identifying optimal strategies to deliver nanomedicine into solid tumors.

## Conflict of Interest

The authors declare no conflict of interest.

## Supporting information

Supplementary

Supplementary

Supplementary
