# Supplementary material for: Therapeutic Remodeling of the Tumor Microenvironment Enhances Nanoparticle Delivery
Source: Adv Sci (Weinh). 2019 Jan 22;6(5):1802070. doi: 10.1002/advs.201802070 (PMC6402396; doi:10.1002/advs.201802070)
Supplement: Supplementary file 1 — Supplementary [file ADVS-6-1802070-s002.pdf]

Copyright WILEY-VCH Verlag GmbH & Co. KGaA, 69469 Weinheim, Germany, 2019.

ADVANCED SCIENCE
Open Access

Supporting Information

for *Adv. Sci.,* DOI: 10.1002/advs.201802070

Therapeutic Remodeling of the Tumor Microenvironment Enhances Nanoparticle Delivery

Yuanxin Chen, Xiujie Liu, Hengfeng Yuan, Zhaogang Yang, Christina A.von Roemeling, Yaqing Qie, Hai Zhao, Yifan Wang, Wen Jiang, and Betty Y. S. Kim**

Copyright WILEY-VCH Verlag GmbH & Co. KGaA, 69469 Weinheim, Germany, 2019.

Supporting Information

Therapeutic remodeling of the tumor microenvironment enhances nanomedicine delivery

Yuanxin Chen, Xiujie Liu, Hengfeng Yuan, Zhaogang Yang, Christina A. von Roemeling, Yaqing Qie, Hai Zhao, Yifan Wang, Wen Jiang, Betty Y. S. Kim**

Experimental Section

Cranial window preparation

The transparent cranial window procedure was adopted from previously published methods with modifcations.[1] Briefly, Six- to 8-week-old C57BL/6J mice were anesthetized by the inhalation of isoflurane (4% for induction; 1.5% to 2% for surgery, and 0.5% to 1.0% for imaging) and immobilized in the stereotactic apparatus (Model942, Kopf Instruments). After anesthesia, dexamethasone and buprenorphine were subcutaneously administered to reduce inflammation and pain, respectively. Body temperature was monitored and maintained at 37.0°C by using a heating blanket (Homeothermic Blanket D+Systems, Harvard Apparatus). Hair was removed to expose the parietal-occipital skull, the scalp was removed, and 5 minutes later a high-speed micro drill (K1040, Foredom) was used to create a 4-mm craniotomy over the primary somatosensory cortex (coordinates: P2, L2) under the dissecting microscope, and a custom-made metal plate was glued onto the skull with dental acrylic cement. After tumor cells were injected into the cortex, a cover slip (5-mm diameter) was added to protect the exposed cortex and to reduce movement artifacts caused by respiration. The antibiotic enrofloxacin (5 mg/kg s.c.) and dexamethasone (0.2 mg/kg s.c.) were injected for 3 days to prevent infection and inflammation. All surgery was performed only if the animals' physiological variables remained within normal limits. All animals were maintained at the animal facility of Mayo Clinic in Florida in specific pathogen-free environment. All animal procedures were approved by the Mayo Clinic Institutional Animal Care and Use Committee (IACUC) and were done in accordance with the National Institutes of Health Guide for the Care and Use of Laboratory Animals.

Tumor cell transfection and injection

Mouse glioma 261 (Gl261) cell lines were transfected with Plasmid mito-dendra2 (#55796, Addgene) by using Lipofectamine 3000 reagent (Invitrogen) as described in the manufacturer's protocol. Briefly, we first plated 5×105 GL261 cells in 6-well plates until cells reached 70%-90% confluence for transfection. Diluted plasmids were then added into diluted Lipofectamine 3000 (1:1 ratio) and incubated for 10-15 min at room temperature, and this complex was added to the 6-well plates to transfect the plated cells. Cells were visualized and analyzed 2-4 days later, and neomycin was added to select the transfected cells every 3 days when the complete DMEM media was replaced (DMEM, 10% fetal bovine serum, 1% penicillin/streptomycin). Then, to further purify the transfected tumor cells, the cells were harvested and purified with a FACS Aria system (Becton Dickinson) at the exponential growth phase.

After the craniotomy surgery, and when the plated tumor cells were 70%-92% confluent, the GL261 cells were harvested and 1.5-2 µL of GL261-dendra2-mito cells (1.5~2×105) were then injected perpendicularly into the exposed cortex (posterior 2 mm, lateral 1.5 mm, and caudal 1 mm) with a microsyringe (32-gauge needles, Hamilton) under stereomicroscopic guidance. The entire injection process lasted 5 minutes, and another 5 minutes was allowed to elapse before the needle was extracted.

Nanoparticle synthesis and characterization

Water-soluble, 655-nm emission carboxy-terminated quantum dots were purchased from Invitrogen. Monofunctional amine-terminated polyethylene glycol (PEG) of molecular weight 10 kD were purchased from Sigma-Aldrich. Conjugation was performed via carbodiimide-mediated chemistry.[2] Briefly, a 1:100 ratio of quantum dots:PEG amine molar mixture was reacted with a 1000:1 molar ratio of N-dimethylaminopropyl-N'-ethylcarbodiimide:quantum dots for 2 h. The finished product was purified on a Sephadex (G-25) column (Sigma-Aldrich). The hydrodynamic diameter and surface charge of purified QD-mPEG10k were characterized by using the Zetasizer Nano ZS90 system (Malvern). Fluorescence intensity measurements were performed using a Spectrofluorometer (Horiba Scientific).

Multi-photon intravital Imaging

An upright laser scanning microscope (BX61WI, Olympus) attached to a Ti:sapphire pulsed laser system (80 MHz repetition rate, <100 fs pulse width, Spectra Physics) and software (Prairie view 5.2, Bruker) was used for two-photon fluorescence imaging. Water-immersion

objectives (20x, NA 1.00, WD 2 mm; and 40x, NA 0.80, WD 3 mm; Olympus) were used for fluorescence imaging in vivo as follows. Mice were anaesthetized and immobilized on a custom-made microscope stage and 890-nm irradiation wavelength was used to generate an second-harmonic generation (SHG) signal from extracellular matrix; the vasculature was visualized with dextran conjugated with Texas Red (20 kD, 40 kD, and 70 kD, 12.5 mg/kg, ThermoFisher) and emission light (nanoparticles, dextran conjugated with Texas Red, Dendra2 protein and extracellular matrix) was distinguished and detected with 660/40, 615/50, 525/50 and 460/50 filters, respectively. Images were taken at 12-bit depths at a resolution of 512 × 512 pixels with a pixel dwell time of 4 μs. The average laser power for imaging was <50 mW.

Treatment protocols

Treatment was begun 2 weeks after orthotopic tumor implantation. Mice were treated with IgG (5 mg/kg i.p., Jackson ImmunoResearch Lab), the anti-mouse VEGFR-2 antibody mAb DC101 (10 mg/kg, Eli Lilly), and a mouse anti-TGFβ antibody (5 mg/kg, 1D11.16.18, BioXCell) every 3 days for a total of three doses (Figure 2a). This dosing regimen was based on previous studies of low-dose anti-angiogenic agents to normalize tumor vasculatures.[3]

In Vivo Nanoparticle Imaging

Mice were sedated via inhaled isofluorane and kept warm on a heating pad. Fluorescent nanoparticles were injected intravenously via the orbital route at a dose of 5 nmol, based on our previous protocol.[4] Images were obtained with a custom-built multiphoton intravital microscope. To measure circulation half-life, baseline intravascular fluorescence intensities before nanoparticle injection were recorded, nanoparticles were injected, and intravascular fluorescence intensities were recorded, normalized, and plotted over time. The half-life was calculated as the time for the total intensity to decrease by 50% from the peak. All fluorescence images were analyzed as described below.

Imaging analysis

All images were processed by using the open-source software Fiji (**http://fiji.sc/**) and a custom-written program with 64-bit Matlab (Version 8.5.0 R2015a, Mathworks). Registration

had been used to perform the intensity-based alignment of images at different time points with the imregister function in Matlab and StackReg plugin in Fiji. Maximum intensity projection (MIP) with 10 slices (10 µm) was used to display vasculature morphology for further analysis.

To measure the permeability of blood vessels, regions of interest (ROIs) were manually outlined within the brain parenchyma. The corresponding vessels, as measured by mean fluorescence intensity of each ROI, were calculated on a minute-by-minute basis. The relative intensity of fluorescence signals in the brain parenchyma was defined as $\Delta F = (F - F0)/F0$, where F and F0 are fluorescence intensity at any given time point and the initial time point, respectively.[5] Permeability surface-area product was used to quantify vasculature permeability.[6]

To measure vessel coverage area, generally we set a threshold value and used some function (e.g., function bwareaopen in Matlab) to segment the vessel while remove the background and small objects. The percentage of vessel coverage was defined as the total number of positive pixels after segmentation divided by the total number of pixels in the original image, and the vessel coverage was equal to the product of the vessel coverage percentage and the real size of the original image.

To measure vessel tortuosity in tumor areas, we defined tortuosity as the arc-chord ratio (L/C),[4] which measures the ratio of vessel length along the central axis (L) to the direct distance between the adjacent vessel branch points (C). Threshold values were chosen to remove the background and segment vessel for L/C measurement.

To measure nanoparticle distribution in an area of brain tumor, custom-written scripts within Matlab were written to extract and segment nanoparticles from tumor areas to measure fractions of coverage area and for correlation analysis of nanoparticle distribution while excluding the vasculature and other artifacts (Supplementary Figure S6). Similarly, the extracellular matrix was segmented for the analysis of area fraction and intensity.

Statistical analysis

Data for all bar graphs were plotted as means and error bars corresponding to standard deviations. Comparisons between three or more independent groups were made with one-way

analysis of variance. Comparisons between two sample groups were made with unpaired non-parametric Mann–Whitney tests. All tests were two-tailed. A *P* value <0.05 was considered statistically significant. Statistical analysis was done with GraphPad Prism 7 software.

References

1. Heo, C.; Park, H.; Kim, Y. T.; Barg, E.; Kim, Y. H.; Kim, S. G.; Suh, M. *Sci. Rep.* **2016**, 6, 27818.
2. Egawa, G. *et al.* Intravital analysis of vascular permeability in mice using two-photon microscopy. *Sci Rep* **2013**, *3*, 1932.
3. Y. Huang, J. Yuan, E. Righi, W. S. Kamoun, M. Ancukiewicz, J. Nezivar, M. Santosuosso, J. D. Martin, M. R. Martin, F. Vianello, P. Leblanc, L. L. Munn, P. Huang, D. G. Duda, D. Fukumura, R. K. Jain, M. C. Poznansky. *Proc. Natl. Acad. Sci. U.S.A.* **2012**, 109, 17561.
4. Jiang, W.; Huang, Y.; An, Y.; Kim, B. Y. S. Remodeling Tumour Vasculature to Enhance Delivery of Intermediate-Sized Nanoparticles. *ACS Nano* **2015**, *9*, 8689–8696.
5. Mittapalli, R. K.; Adkins, C. E.; Bohn, K. A.; Mohammad, A. S.; Lockman, J. A.; Lockman, P. R. Quantitative Fluorescence Microscopy Measures Vascular Pore Size in Primary and Metastatic Brain Tumors. *Cancer Res*. **2017**, *77*, 238-246.
6. Kim, B. J.; Yang, E.; Kim, N. Y.; Kim, M. J.; Kang, D. W.; Kwon, S. U.; Kim, J. S. Vascular Tortuosity May Be Associated With Cervical Artery Dissection. *Stroke*, **2016**, *47*, 2548-2552.

Supplementary Figures

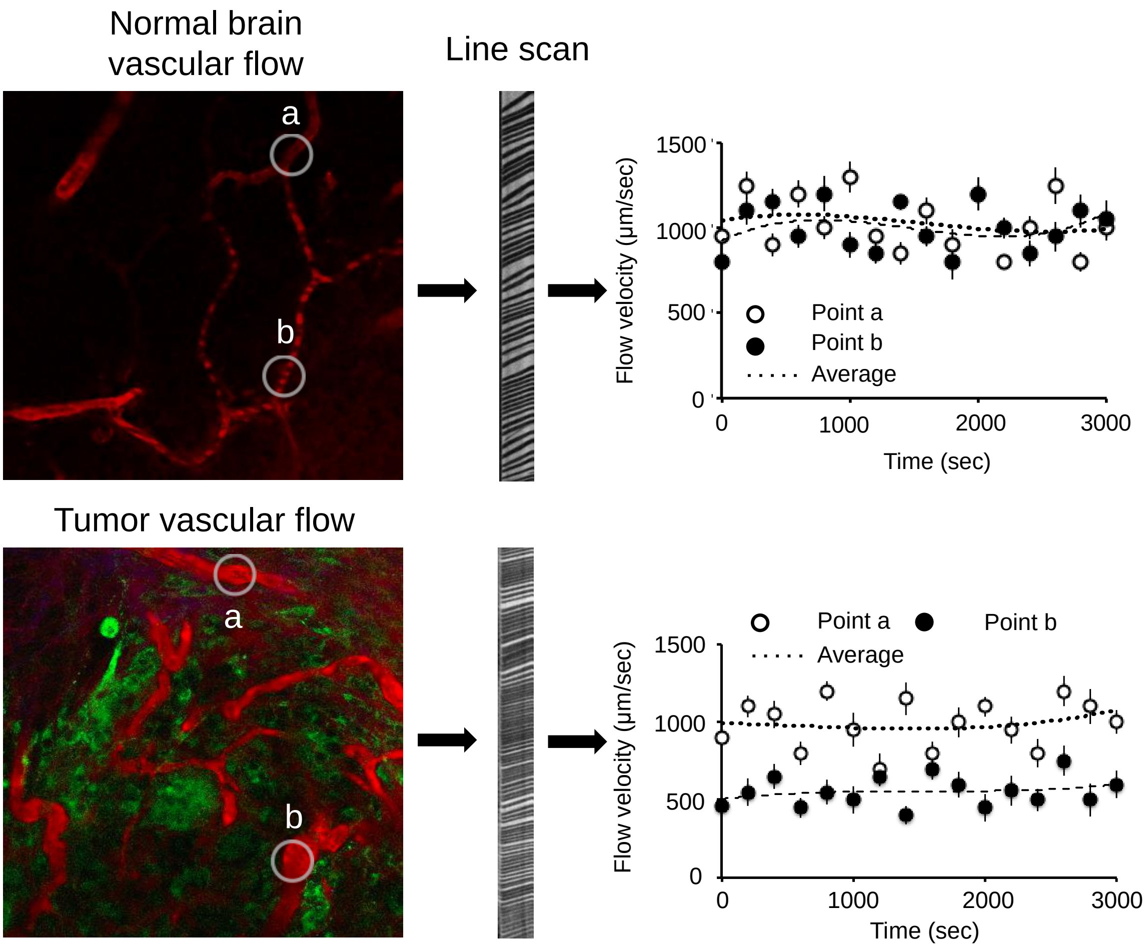

Figure S1. Intravital microscopy revealed heterogeneous vascular flow within tumor blood vessels relative to vessels within normal brain using a line-scan technique. Excessive production of extracellular matrix proteins, hyperproliferation of tumor cells, and elevated interstitial fluid pressure cause compression of tumor vessels, leading to flow stasis in certain areas while maintaining adequate perfusion in others.

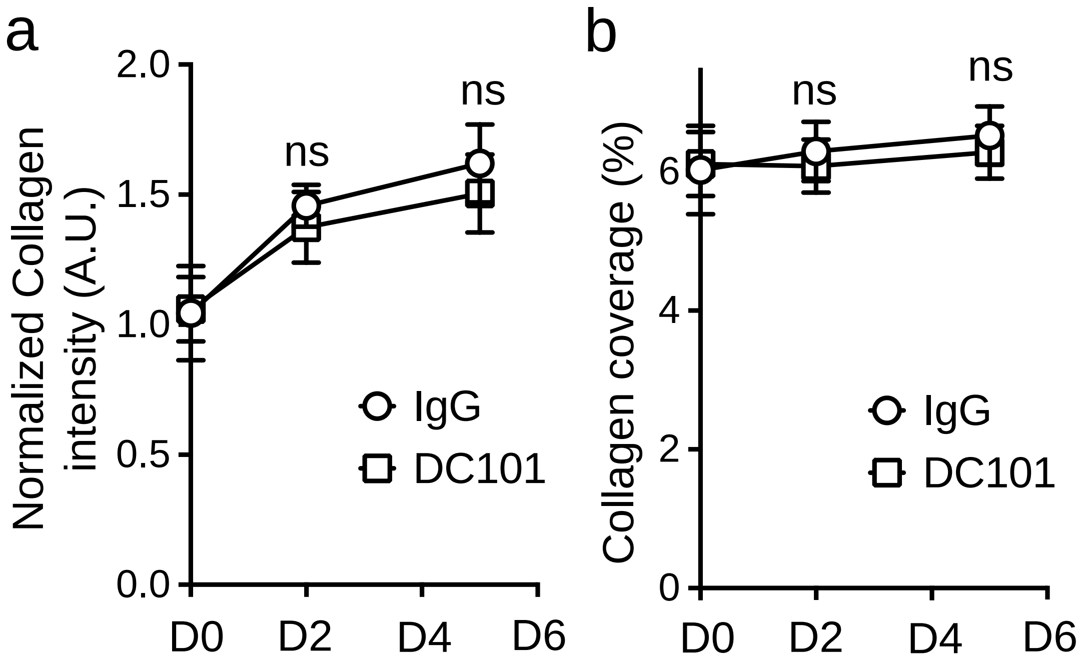

Figure S2. DC101 treatment alone did not result in a significant decrease in extracellular matrix content as measured by (a) collagen deposition density and (b) total proportion of collagen coverage relative to IgG treated controls in GL261 glioma. Error bars: mean ± SD; n = 4; ns: p>0.05.

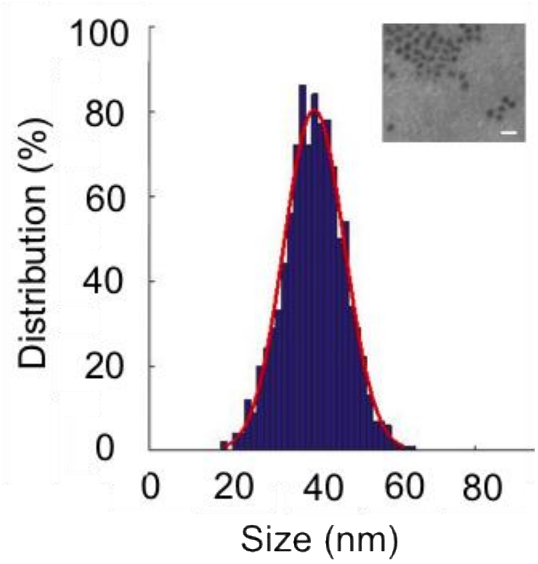

Nanoparticle	Hydrodynamic diameter (nm)	Surface Charge	Blood half-life (min)	Polydispersity index (PDI)
QD-PEG-10K	39.5±3.3	-2.8±0.41mV	180	0.2

Figure S3. Characterization of polyethylene glycol (PEG)-modified water-soluble semiconductor nanocrystals (QDs). Inset: TEM image of QD-PEG-10K.

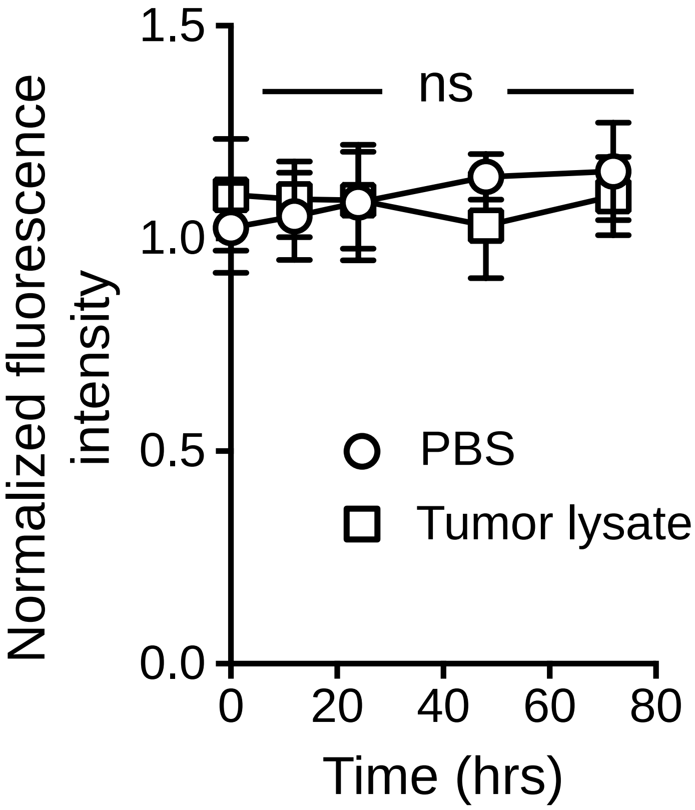

Figure S4. The polyethylene glycol (PEG) -modified water-soluble semiconductor nanocrystals (QDs) maintained their fluorescence intensity within biological environment for prolonged periods, allowing quantitative tracking of particle distribution within tissues. PBS, phosphate-buffered saline. Error bars = mean ± SD; n = 3; ns: p>0.05.

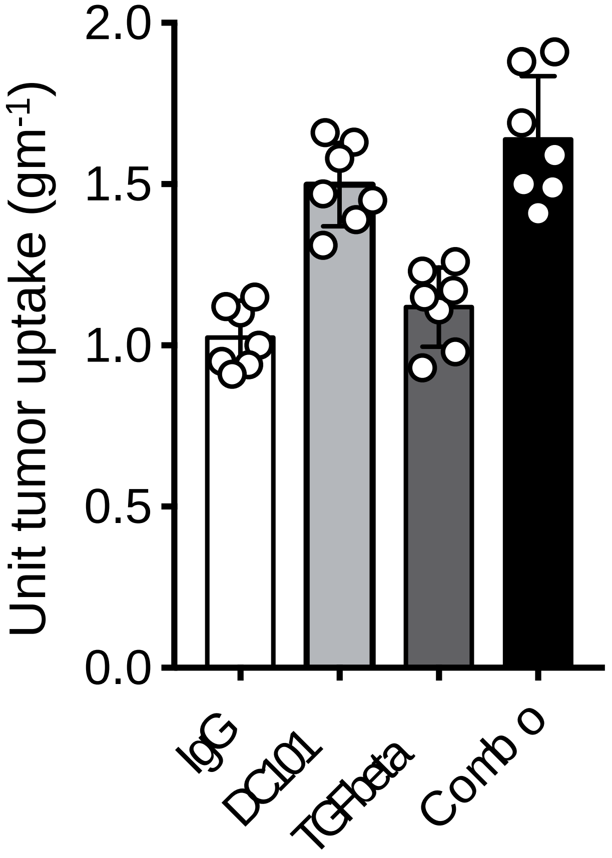

Figure S5. Quantification of nanoparticle uptake under different treatment conditions. Tumor uptake was quantified indirectly by using total fluorescence intensity as described in Supplementary Methods. Treatment with DC101 led to improved nanoparticle delivery relative to treatment with IgG. No difference was found in nanoparticle uptake by tumors in combined treatment versus DC101 alone. Error bars = mean ± SD.

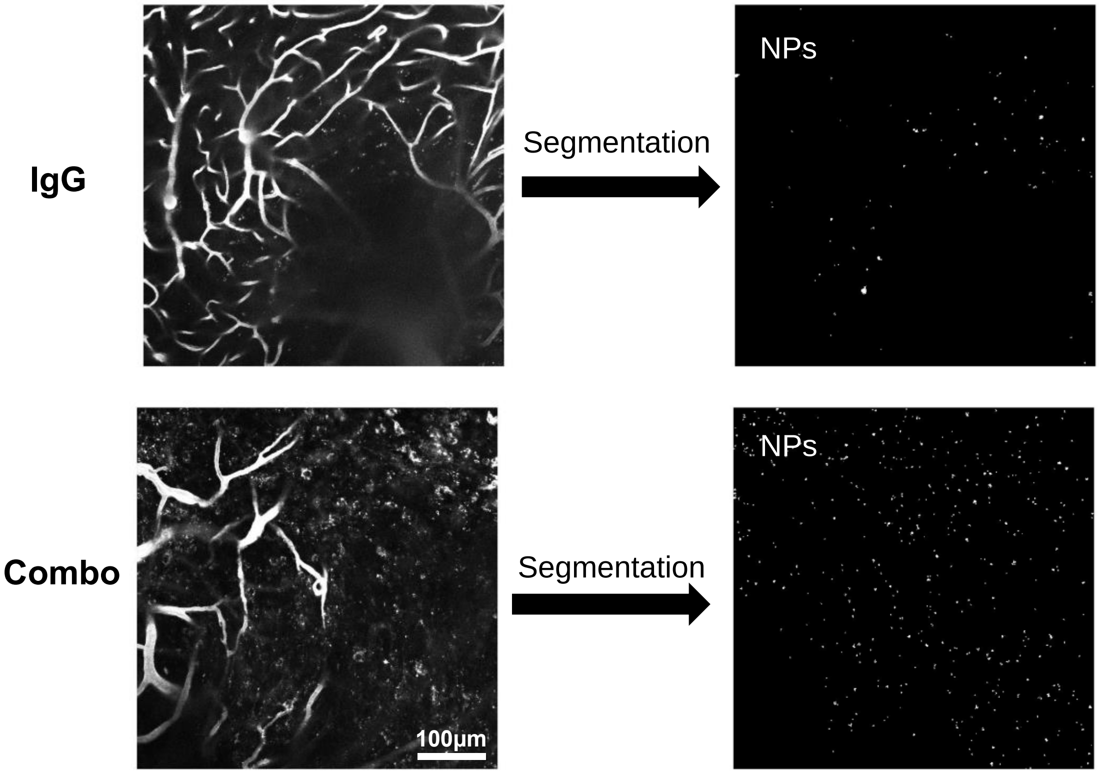

Figure S6. Auto-segmentation algorithms were developed to quantify nanoparticle (NP) accumulation within tumor tissues.
